# Factors Associated with Utilization of Modern Contraceptives among Female Undergraduates of Addis Ababa University Ethiopia

**DOI:** 10.4314/ejhs.v35i2.7

**Published:** 2025-03

**Authors:** Eskinder Kebede

**Affiliations:** 1 Addis Ababa University, School of Medicine, Department of Gynecology and Obstetrics

**Keywords:** Addis Ababa University, Contraceptives, Modern contraceptives, Utilization, Ethiopia

## Abstract

**Background:**

The prevalence of unintended pregnancy is very high in Addis Ababa. The lack of knowledge about modern contraception methods and poor utilization are the main contributing factors to the increasing prevalence of unintended pregnancies. In this study, we aimed to determine the level of and assess factors affecting the utilization of modern contraceptive methods.

**Methods:**

An institution-based cross-sectional study was conducted at Addis Ababa University in 2022. A multi-stage sampling technique was used to select the students. Data were collected through a self-administered questionnaire and analyzed using descriptive statistics. Chi-square tests and logistic regression analysis were used to identify predictors of the outcome variable.

**Results:**

A total of 691 students were included in the study, with a mean age of 20.8 ± 1.3 years. More than half of the participants (56%) had ever taken sexual education lessons, and 114 (16.5%) were currently sexually active. The ever use of modern contraceptives was 19.4%, and the current use of modern contraceptives was 19.0%. Factors strongly associated with the utilization of modern contraceptives include participants' age (AOR: 0.176, 95% CI: 1.150, 4.117), field of study (medical school) (AOR: 3.501, 95% CI: 1.475, 8.309), being in the first and second year of study (AOR: 0.270, 95% CI: 0.133, 0.548), and having taken sexual education lessons (AOR: 3.413, 95% CI: 1.771, 6.579).

**Conclusion:**

Modern contraceptive use is low (19.4%). This study found that age, field of study, year of study, taking sexual education lessons, and ever use of emergency contraception are strongly associated with the utilization of modern contraception among female undergraduate students. School health education should be emphasized.

## Introduction

Unintended pregnancy occurs when a woman does not desire children at the time of conception or when a pregnancy occurs earlier than planned; it can be classified as either unwanted or mistimed. It has significant health impacts on both the mother and the newborn. The rate of unintended pregnancies and unintended births remains high, at 44% and 23%, respectively. Of all pregnancies worldwide, 21.6 million occurred in Africa, with 8.85 million in Eastern Africa (1, 2).

Studies have shown that non-use, misuse, and lack of knowledge about contraceptive methods are among the major causes of unintended pregnancy worldwide, with knowledge of emergency contraception being strongly linked to the reduction of unintended pregnancies (3, 4). Modern contraceptive methods, defined as products or medical procedures that prevent pregnancy following sexual intercourse, are the most effective means of preventing unintended pregnancies (3).

The prevalence of unintended pregnancy in sub-Saharan Africa is very high (30.01%, 95% CI: 29.38–30.74), with young people and adolescents being particularly affected (5). The lack of knowledge about modern contraception and poor utilization contribute significantly to the increasing prevalence of unintended pregnancies.

In Ethiopia, the national rate of unintended pregnancy is 38%. University students are among the most vulnerable groups for unintended pregnancies, with studies showing a high prevalence in this demographic (6). This situation calls for policymakers and health professionals to provide comprehensive reproductive health and contraception services to address the unmet needs of university students (6, 7). Effective contraception has been shown to reduce the burden of various reproductive health issues globally, including unwanted pregnancies. However, there has been a reduction in the usage of modern contraceptives in some parts of the world, with sub-Saharan Africa showing the lowest usage rates (8). There are several factors contributing to the low utilization of contraceptives, including lack of knowledge, personal beliefs, need for parental consent, fear of side effects, and religious views (9, 10).

In Ethiopia, various studies have assessed university students' knowledge of modern contraceptives. While the knowledge level was fair to optimal, the utilization of these methods was very low (11, 12).

Several factors contribute to the poor utilization of modern contraceptives in Africa. A study conducted in Nigeria found that religious beliefs and fear of side effects were the most common reasons for low contraceptive use, accounting for 70% of the non-usage (13). Similarly, a study in Aisaita, Ethiopia, found that marital status, age, residence, students' living conditions, and financial resources were significantly associated with the use of modern family planning methods (14).

Modern contraceptives, including female and male sterilization, intrauterine devices (IUDs), hormonal methods (oral pills, injectables, implants), condoms, and vaginal barrier methods, are safe and effective in preventing pregnancy and sexually transmitted infections. The utilization of modern contraceptives refers to the use of these methods either at any point in the past (ever use) or currently (current use) (15).

This study aims to assess the level of modern contraception utilization, awareness, and the factors affecting its use among female undergraduate students at Addis Ababa University, one of the most vulnerable groups in society. The objective is to determine the level of and assess factors associated with the utilization of modern contraceptive methods among female students.

## Materials and Methods

This study employed an institution-based crosssectional design conducted at Addis Ababa University (AAU) in 2022. AAU, established in 1950 as the University College of Addis Ababa (UCAA), is the oldest and largest higher education institution in Ethiopia. In the 2019/2020 academic year, the university had 48,673 students (33,940 undergraduates, 13,000 master's students, and 1,733 PhD students) and 6,043 staff members (2,408 academics and 3,635 support staff) across 14 campuses. Currently, AAU has 13,489 regular undergraduate students, 3,528 of whom are female (16).

The study population consisted of 3,351 female undergraduate students at AAU in 2022. A two-stage sampling method was employed to select participants. Initially, participants were chosen from each of the university's 11 colleges, with allocation proportional to the size of each college. Subsequently, participants were selected from each college, proportional to their year of study, using simple random sampling.

The sample size was determined using a simple proportion formula for cross-sectional studies in Epi Info software, based on a proportion of 68.4% of students having good knowledge (17). With a margin of error of 4% and a 95% confidence interval, the final sample size was calculated to be 706.

**Inclusion criteria**: Female undergraduate students currently enrolled at AAU and willing to participate in the study.

**Exclusion criteria**: Students not enrolled in regular programs or absent during data collection.

Data were collected via a self-administered questionnaire in English, as the university's official language of instruction is English. The questionnaire was adapted from a similar study and pretested on students from a different university. Data were entered, edited, and cleaned using IBM SPSS version 21 for statistical analysis. Descriptive statistics such as proportions, percentages, and frequency distributions were calculated. A Chisquare test was used to assess correlations between independent and dependent variables. For variables showing a correlation, bivariate logistic regression was conducted to assess statistical associations. Variables with p-values less than 0.25 were entered into multivariable logistic regression to control for confounders. Statistical significance was considered for p-values less than 0.05, with odds ratios (ORs) and 95% confidence intervals (CIs) calculated.

Ethical approval was obtained from the Institutional Review Board (IRB) of the Addis Ababa University School of Medicine. Informed consent was obtained from all participants, with assurances of anonymity and confidentiality throughout the study. Participants were informed of their right to refuse or withdraw from the study at any time.

## Results

A total of 691 participants were included in the study, with a mean age of 20.8 ± 1.3 years. Most participants (66.1%) were Orthodox Christians, and 93.2% had no partner (boyfriend or husband). A significant portion (83.5%) were not sexually active. Only 387 (56%) participants had ever received sexual education, and 172 (24.9%) had ever used emergency contraception (Table 1).

**Table 1:** Sociodemographic & Reproductive characteristics of Addis Ababa University female undergraduate students, 2022 (n=691)

Variable	Category	Frequency (n = 691)	Percent
Age group (year)	≤20	329	47.8
	>20	362	52.4
Religion	Christian	615	89.0
	Muslim	76	11.0
Marital status	No partner	644	93.2
	With partner	47	6.8
Field of study	Medical	352	30.8
	Non-medical	339	69.2
Year of study	Year I	241	34.9
	Year II	241	34.9
	Year III and above	209	30.2
Currently sexually active	Yes	114	16.5
	No	577	83.5
Past sexual education lesson	Yes	387	56.0
	No	304	44.0
Ever used Emergency Contraception	Yes	172	24.9
	No	519	75.1
Ever heard about modern contraception	Yes	489	70.8
	No	202	29.2

The ever use of modern contraceptives was 19.4%, and the current use was 19.0%. The main reasons for non-use included not being sexually active (79.1%), fear of side effects, and lack of knowledge about contraceptive methods (Table 2).

**Table 2:** Utilization of modern contraception of Addis Ababa University female undergraduate students, 2022 (n=691)

Variable	Frequency (n = 691)	Percent
Ever heard about modern contraception (691)		
Yes	489	70.8
No	202	29.2
Information obtained from (n = 489)		
Media	222	45.3
School	189	38.6
Friends and family	39	7.9
Health facility	39	7.9
Methods ever heard		
Condoms	463	94.6
Pills	427	87.3
Injectable	402	82.2
Emergency contraception	370	75.6
IUCD	220	44.9
Implant	104	21.2
Where can a women obtain modern contraception(691)		
Pharmacy	434	62.8
Health facility	112	16.2
I don't know	145	21.0
Ever use of Modern contraception(n = 489)		
Yes	95	19.4
No	375	80.6
Current use of modern contraception(n = 489)		
Yes	93	19.0
No	396	81.0
Reason for not use*		
I am not sexually active currently	387	79.1
I don't need now	100	20.4
I don't know details of use	75	15.3
Fear of side effect	65	13.2
I don't know	60	12.2
I am using traditional methods	4	0.8

More than one answer is possible

Logistic regression analysis revealed that age, field of study, year of study, taking sexual education lessons, and ever use of emergency contraception were strongly associated with modern contraceptive utilization. Participants under 20 years old were twice as likely to use modern contraceptives. Those in medical schools had 3.5 times the odds of using modern contraceptive methods, and those who had received sexual education had 3.5 times the odds of utilizing contraceptives (Table 3)

**Table 3:** The bivariate and multivariate binary regression test between selected explanatory variables and utilization of modern contraception methods of Addis Ababa University female undergraduate students, 2022 (n = 489)

Variable	Yes (%)	No(%)	P value	AOR (95%CI)	P value
Age					
≤20	40	170	0.854	2.176 (1.150, 4.117)*	P<0.017
>20	55	224		1	
Religion					
Christian	86	353	0.778	0.754(0.320, 1.777)	P<0.320
Muslim	9	41		1	
Marital status					
No partner	85	368	0.192	0.562(0.212, 1.487)	P<0.212
Has partner	10	26		1	
Field of study					
Medical	10	141	0.000	3.501(1.475, 8.309)**	P<0.004
Non-medical	85	253		1	
Year of study					
Year I	38	109	0.000	0.137(0.060, 0.312)***	p<0.001
Year II	40	128	0.000	0.270(0.133, 0.548)***	p<0.001
Year III and above	17	157		1	
Have you ever had sexual education lesson					
Yes	59	263	0.444	3.413(1.771, 6.579)***	p<0.001
No	34	126		1	

## Discussion

This study found that 16.5% of participants were currently sexually active, a rate lower than studies in Dilla and universities in the Amhara region (21% and 31.2%, respectively), but higher than a study in Nigeria (11%). This difference may be due to variations in data collection timing (12, 17).

The study found that modern contraceptive use was low (19.4%), which is consistent with studies from Nigeria (16%) and Amhara universities (15.6%) but lower than those from Dilla, Uganda, and Tanzania (46.6%–64%) (12, 15).

The most common reasons for not using modern contraceptives were being sexually inactive and fear of side effects, which mirrors findings from Dilla. However, it differs from studies in Tanzania and Zanzibar, where religious beliefs were the primary reason for non-use (9, 10).

Our study also showed that age, field of study, year of study, and prior exposure to sexual education were strongly associated with contraceptive utilization, in contrast to findings in other studies (11, 18). The media was the most common source of information on contraceptives (45.3%), with health workers playing a minimal role (13).

As this study used cross-sectional data from a single university, causal relationships cannot be inferred. Additionally, social desirability bias may have influenced the responses despite efforts to minimize it.

In conclusion, the use of modern contraception among female university students is low. Improved sexual and reproductive health education and communication are urgently needed. Universities should incorporate these issues into curricula, and public health interventions, such as behavior change communication, should target the factors influencing contraceptive utilization. Further qualitative research is needed to explore the barriers to contraceptive use in Ethiopia.

## References

[1] Merga Jaleta, Wirtu Desalegn, Bekuma Tariku Tesfaye, Regasa Misganu Teshoma (2021). Unintended pregnancy and the factors among currently pregnant married youths in Western Oromia, Ethiopia: A mixed method. PLoS One.

[2] Beyene Girma Alemayehu (2019). Prevalence of unintended pregnancy and associated factors among pregnant mothers in Jimma town, southwest Ethiopia: a cross sectional study. Contracept Reprod Med.

[3] Hubacher D, Trussell J (2015). A definition of modern contraceptive methods. Contraception.

[4] Goldsmith KA KL, Rosenberg KD, Sandoval AP, Lapidus JA (2008). Unintended childbearing and knowledge of emergency contraception in a population-based survey of postpartum women. Maternal Child Health J.

[5] Tebekaw Yibeltal, Aemro Bezuhan, Teller Charles (2014). Prevalence and determinants of unintended childbirth in Ethiopia. BMC Pregnancy Childbirth.

[6] Ayalew Hiwotie Getaneh, Liyew Alemneh Mekuriaw, Tessema Zemenu Tadesse (2022). Prevalence and factors associated with unintended pregnancy among adolescent girls and young women in sub-Saharan Africa, a multilevel analysis. BMC Womens Health.

[7] Ethiopia CSAAA (2021). EDHS.

[8] Gakidou E VE (2007). Use of Modern Contraception by the Poor Is Falling Behind. PLOS Med.

[9] Kara WSK BM, Mao J (2019). Knowledge, Attitude, and Practice of Contraception Methods Among Female Undergraduates in Dodoma, Tanzania. Cureus.

[10] Dimoso PJ MM SM (2014). The Use of Contraceptives among Female Students in State University of Zanzibar. Food and Public Health.

[11] Simegn Amare, Tiruneh Dawit, Seid Tigist, Ayalew Florence (2020). Contraceptive Demand, Utilization and Associated Factors Among University Female Students in Amhara Region, Ethiopia: Institution-Based Cross-Sectional Study. Open Access J Contraception.

[12] Soressa Masresha, Astatkie Ayalew, Berhane Yemane, Mitiku Shimelis (2016). Contraceptive Use and Associated Factors among Dilla University Female Students, Southern Ethiopia. Journal of Marketing and Consumer Research.

[13] Adedini Sunday A, Babalola Stella, Ibeawuchi Charity, Omotoso Olukunle, Akiode Akinsewa, Odeku Mojisola (2018). Role of Religious Leaders in Promoting Contraceptive Use in Nigeria: Evidence From the Nigerian Urban Reproductive Health Initiative. Global Health: Science and Practice.

[14] Mahamed Keder, Medhanyie Araya Abrha, Liben Misgan Legesse, Shamie Reda (2017). MODERN FAMILY PLANNING METHODS UTILIZATION AND ASSOCIATED FACTORS AMONG FEMALE STUDENTS IN AYSAITA TOWN, NORTHEASTERN ETHIOPIA. Med. res. chronicles [Internet].

[15] Tsegaw Menen, Mulat Bezawit, Shitu Kegnie (2022). Modern Contraceptive Utilization and Associated Factors Among Married Women in Liberia: Evidence from the 2019 Liberia *Demographic and Health Survey*. Open Access J Contracept.

[16] University AA (2021). Seek Wisdom, Elevate your Intellect and Serve Humanity [Internet].

[17] Arowojolu AO IA, Roberts OA, Okunola MA (2002). Afr J Reprod Health. 2002 Aug. Sexuality, contraceptive choice and AIDS awareness among Nigerian undergraduates. Afr J Reprod Health.

[18] Nsubuga H SJ, Sempeera H, Makumbi FE (2016). Contraceptive use, knowledge, attitude, perceptions and sexual behavior among female University students in Uganda: a crosssectional survey. BMC women's health.

[19] Ahmed Zainab Datti (2017). Knowledge and utilization of contraceptive devices among unmarried undergraduate students of a tertiary institution in Kano State, Nigeria 2016. Pan African Medical Journal.

